# Serum TNFα levels at 24 h after certolizumab pegol predict effectiveness at week 12 in patients with rheumatoid arthritis from TSUBAME study

**DOI:** 10.1186/s13075-021-02547-2

**Published:** 2021-06-01

**Authors:** Yusuke Miyazaki, Kazuhisa Nakano, Shingo Nakayamada, Satoshi Kubo, Shigeru Iwata, Kentaro Hanami, Shunsuke Fukuyo, Ippei Miyagawa, Ayako Yamaguchi, Akio Kawabe, Kazuyoshi Saito, Yoshiya Tanaka

**Affiliations:** 1 The First Department of Internal Medicine, School of Medicine, University of Occupational and Environmental Health, 1-1 Iseigaoka, Yahata-nishi, Kitakyushu, 807-8555 Japan; 2 Tobata General Hospital, Kitakyushu, Japan

**Keywords:** Rheumatoid arthritis, Biological therapies, DMARDs, Pharmacology, Biomarkers

## Abstract

**Objective:**

To estimate the relationship between serum TNFα, IL-6, and serum CZP levels and the clinical response to CZP in RA patients in the TSUBAME study.

**Methods:**

One hundred patients with RA who received CZP were enrolled and multiple clinical parameters, serum TNFα, IL-6, and CZP levels, were assessed at 0, 24, and 48 h and 12 weeks after first administration of CZP.

**Results:**

The CZP therapy significantly improved the DAS28(ESR) at 12 weeks. Serum TNFα and IL-6 levels significantly decreased from baseline at 24 h after the first administration of CZP. Serum TNFα levels at baseline were not related to clinical parameters at baseline and improvement in DAS28(ESR) at week 12 of the CZP therapy. However, serum levels of CZP at 24 h were strongly and negatively correlated with TNFα levels at 24 h, which were negatively correlated with improved rate in DAS28(ESR) at week 12. Only serum levels of TNFα, but not IL-6, at 24 h had a negative correlation with achievement of DAS28(ESR)<2.6 at week 12 by the multivariate analysis (odds ratio 0.01, 95% confidence interval 0.04e−2–0.22, *p* < 0.01). A receiver operating characteristic analysis was conducted to estimate the achievement of DAS28(ESR)<2.6 at week 12 after the CZP therapy and cut-off value of 0.76 pg/ml for serum levels of TNFα at 24 h was yielded (area under the curve=0.75). DAS28(ESR)<2.6 was achieved at week 12 significantly more patients with lower serum TNF levels (≦0.76 pg/ml) at 24 h than those with higher TNF levels.

**Conclusions:**

CZP was highly effective in RA patients who had low serum TNFα levels at 24 h after the initial administration of CZP. Therefore, we propose that serum TNFα levels at 24 h could serve as a biomarker predicting effectiveness to CZP at week 12 in patients with RA.

**Trial registration:**

Clinical trial registration number: UMIN ID:000022831

**Supplementary Information:**

The online version contains supplementary material available at 10.1186/s13075-021-02547-2.

## Background

Rheumatoid arthritis (RA) is a systemic inflammatory disease causing progressive joint destruction and irreversible functional impairmen t[1–3]. Tumor necrosis factor alpha (TNFα) plays critical roles in RA pathology, such as promoting osteoclast differentiation and increasing matrix metalloproteinase (MMP) productio n[4]. TNFα inhibitors suppress arthritis and bone destruction caused by RA and markedly improve RA prognosi s[5, 6]. Certolizumab pegol (CZP), a TNFα inhibitor, is a biological product consisting of a Fab fragment of humanized anti-human TNFα monoclonal antibody conjugated to polyethylene glycol, and it neutralizes membrane and soluble TNF α[7]. In RA patients, early diagnosis and prompt initiation of intensive treatment can improve disease activity early, leading to inhibition of joint destructio n[8, 9]. Therefore, a fast-acting drug that can achieve earlier remission is a superior treatment for RA. A high blood concentration of CZP can be rapidly achieved using a loading dose, which can be maintained by subcutaneous injection administered every 2 week s[10].

In two CZP clinical studies, RAPID1 and J-RAPID, the rate of achieving American College of Rheumatology 20% improvement criteria (ACR20) at 1 week after the first dose of CZP was significantly higher than that in patients receiving placeb o[11, 12], suggesting that CZP has immediate effects. However, the drug’s rapid action has not been verified in actual clinical settings. Moreover, no studies have focused on the onset of action within 1 week after the first dose. To increase RA remission rates, it is necessary to determine the effectiveness of the targeted synthetic disease-modifying antirheumatic drug (DMARD) used at an early point in disease activity, along with measuring the onset of action, and consider switching to another targeted synthetic DMARD if needed. Suitable biomarkers are required to measure these parameters in the clinical setting.

Here, registered as the Anti-TNF Study Utilizing Biomarker Assays to Monitor Early Response to Certolizumab Pegol (TSUBAME study), we prospectively enrolled patients who were treated with CZP in our institution to evaluate its effectiveness and safety starting at 24 h after the first dose in clinical settings, while recording blood CZP concentrations and biomarkers over time to examine their correlation with clinical effects. This study was registered at the UMIN Clinical Trial Registry as UMIN000022831.

## Methods

### Patients and study design

Patients were recruited from the FIRST registry, a registry study of RA patients receiving molecularly targeting antirheumatic drugs at multiple institutions affiliated to our university hospital, the key station, and they were included in this present study. RA was diagnosed when patients met the 2010 American College of Rheumatology (ACR)/European League Against Rheumatism classification criteria or the 1987 ACR classification criteria [13, 14].

TSUBAME is a single-center, single-arm, prospective observational study approved by the ethics review committee of the University of Occupational & Environmental Health, Japan (#H26-200). RA patients aged ≥ 20 years who received CZP between June 2016 and November 2018 were prospectively enrolled in the TSUBAME study. The date of registration of the clinical study was on June 13, 2016.

The inclusion criteria were in accordance with the Guideline for the use of TNF inhibitors in rheumatoid arthritis (RA) (2014 revised version) as follows; RA patients with residual high disease activity (tender joint count ≥ 6 + swollen joint count ≥ 6 + CRP ≥ 2.0 mg/dL or ESR ≥ 28 mm/h) despite adequate use of MTX or MTX in combination with other biological drugs, patients with progressive bone erosion observed using X-ray, or patients with DAS28-ESR ≥ 3.2 using MTX were included in the study.

The following were excluded from the study: (1) patients with serious infections (e.g., sepsis, pneumonia, hepatitis B), (2) patients with active tuberculosis, (3) patients with a history of hypersensitivity to any of the ingredients of the drugs used in this study, (4) patients with or without a history of demyelinating diseases (e.g., multiple sclerosis), (5) patients with congestive heart failure, (6) patients contraindicated for TNF inhibitors in the Guideline for the use of TNF inhibitors in rheumatoid arthritis (RA) (2014 revised version) by the Japan College of Rheumatology, and (7) other patients judged by the investigator to be inappropriate for the study.

CZP was used following the dosage and administration approved within the coverage of Japanese national health insurance (Observation period: 12 weeks).

The mean value of DAS28-ESR prior to the administration of CZP was 5.43 ± 1.40. With a difference of 0.6, an α error of 0.05, and a power of 0.9, the minimum sample size is of 60 patients. The sample size was of 100 patients because of an expected dropout rate of 30%, which was calculated based on the use of different drugs and the feasibility of enrolling for 1 year.

### Treatment with certolizumab pegol

CZP was introduced to patients with RA whose disease activity could not be controlled with antirheumatic drugs. CZP was administered at 400 mg at weeks 0, 2, and 4, followed by 200 mg every 2 weeks. In principle, CZP should be administered subcutaneously at a dose of 200 mg every 2 weeks. However, if symptoms are stable and the patient desires, subcutaneous injection may be administered at a dose of 400 mg at 4-week intervals.

### Clinical effectiveness and treatment outcomes

The primary endpoint measured was the change from baseline in disease activity assessed using the 28-joint count disease activity score-erythrocyte sedimentation rate (DAS28-ESR )[15] up to 12 weeks after the first dose of CZP. Secondary endpoints measured were serum biomarker concentrations (TNFα, interleukin (IL)-6) at baseline, 24 h, and 48 h after the first dose of CZP, changes in serum CZP concentration at 24 and 48 h after the first dose of CZP, and the relationship between the serum biomarkers and serum CZP concentrations and the clinical effectiveness at 12 weeks after CZP initiation.

### Measurement of serum TNFα, IL-6, and CZP levels

Serum TNFα was measured by enzyme-linked immunosorbent assay (ELISA) (R&D SYSTEMS), serum IL-6 was measured by chemiluminescent enzyme immunoassay assay (Fujirebio), and CZP levels were analyzed by ELISA (ImmunnoGuide) according to the manufacturers’ protocols.

### Statistical analysis

Patient characteristics were expressed as mean ± standard deviation. The Kaplan-Meier method was used to assess the retention rates. A paired t test was used to detect differences in disease activity. Univariate logistic regression analysis was performed to determine variables associated with DAS28-ESR remission. Multivariable logistic regression was performed to control potential confounding factors and determine the independent contribution of variables to DAS28-ESR remission. To avoid multicollinearity, Pearson’s correlation co-efficient was calculated between the independent variables to check for multicollinearity problems (*r* > 0.9). All reported *P* values were two-sided and were not adjusted for multiple testing. Differences between groups were considered to be statistically significant at *P* < 0.05. All analyses were conducted using JMP version 12.0 (SAS Institute Inc., Cary, NC).

## Results

### Baseline characteristics of patients with rheumatoid arthritis in the TSUBAME study

The TSUBAME study included 100 RA patients who received CZP and consented to participate. Patient characteristics are shown in Table 1. Approximately 70% of the cases were biological DMARD-naïve. All patients were receiving MTX at baseline, and the median dose was 14 mg/w. Glucocorticoids (GC) were used concomitantly in eight patients, and the median dose was 4.5 mg. The mean DAS28-ESR was 5.4, indicating that most patients had high disease activity.

**Table 1:** Baseline characteristics of patients with RA in the TSUBAME study

Variables	*n* = 100
Age (y)	58 (48–68)
Gender, n (% female)	82 (82.0%)
Disease duration (mo)	29 (8–84)
Stage (I/II/III/IV %)	40/48/6/6
Treatment history	
MTX use at baseline, n (%)	100 (100%)
Dose, mg/w	14 (10–16)
Glucocorticoid use at baseline, n (%)	8 (8%)
Dose, mg/day	4.5 (2.0–7.0)
bDMARDS naïve, n (%)	73 (73%)
Prior use of TNFi	18 (18%)
Prior use of Non-TNFi	6 (6%)
Prior use of both TNFi and non-TNFi	3 (3%)
28-tender joint count	8 (4–13)
28-swollen joint count	7 (3–11)
GH, VAS 0-100 mm	48 (26–70)
EGA, VAS 0-100 mm	40 (28–60)
Pain, VAS 0-100 mm	52 (28–72)
DAS28-ESR	5.4 ± 1.3
HAQ-DI	1 (0.5–1.5)
EQ-5D	0.6 (0.5–0.7)
CRP (mg/dl)	0.6 (0.1–2.1)
ESR (mm/h)	37 (21–68)
Rheumatoid factor (U/ml)	56.4 (21.1–129.1)
Anti-CCP antibody (U/ml)	95.0 (24.7–384.5)
MMP-3 (ng/ml)	87.3 (34.5–328)

Data are mean ± SD, median (IQR), or number (%) of patients

*MTX* methotrexate, *bDMARDS* biological disease-modifying anti-rheumatic drugs, *TNFi* TNFα inhibitor, *GH VAS* patient’s global assessment of disease activity visual analog scale, *EGA VAS* evaluator global assessment of disease activity visual analog scale, *DAS* disease activity score, *HAQ-DI* health assessment questionnaire disability index, *EQ-5D* EuroQol 5 Dimension, *CRP* C-reactive protein, *ESR* erythrocyte sedimentation rate, *MMP-3* matrix metalloproteinase 3

### Clinical effectiveness and changes in serum biomarker and CZP levels

**Fig. 1:**
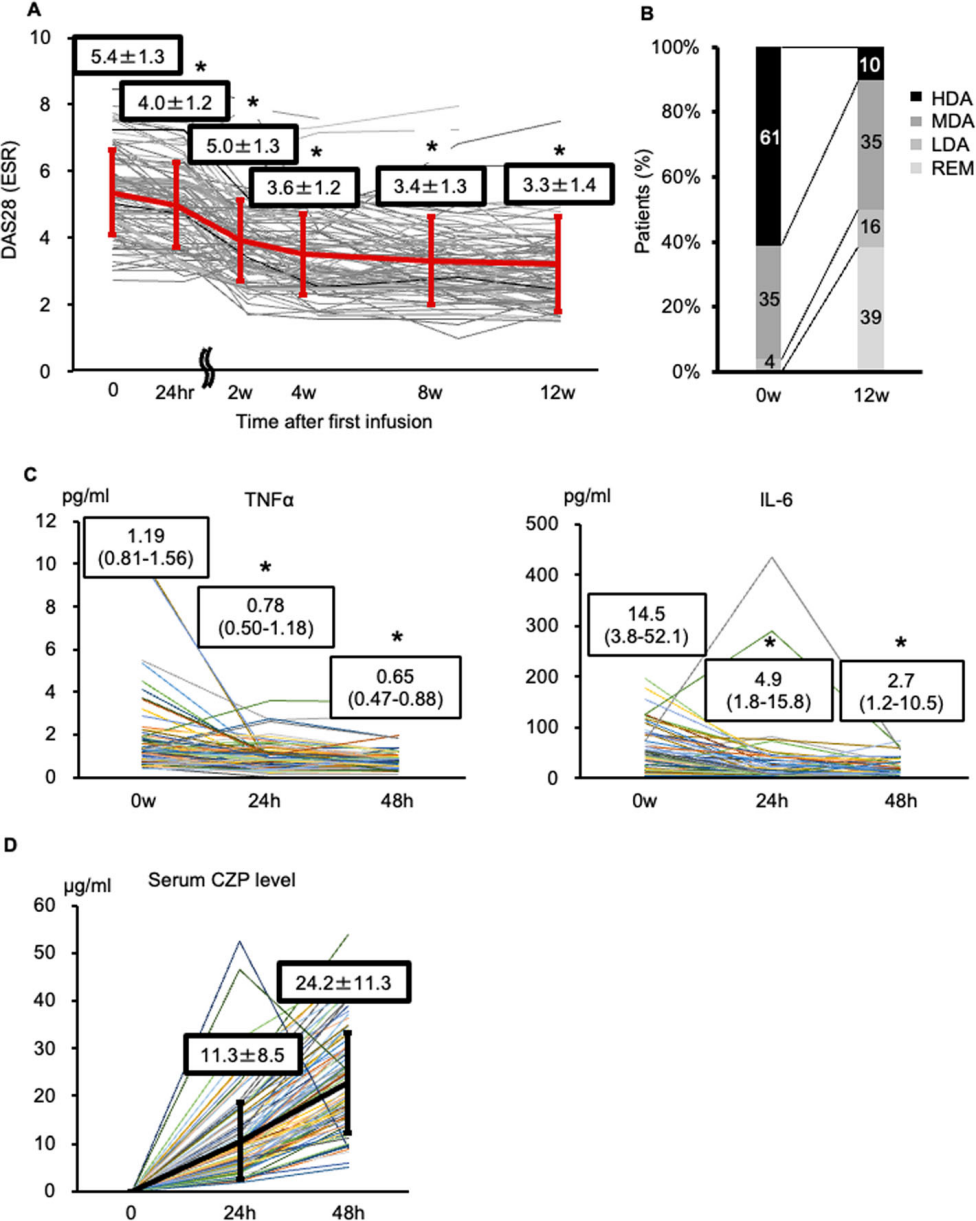
Changes in disease activity, serum biomarker, and CZP levels. **A** Changes in the DAS28(ESR) from baseline to 12 weeks after initiation of CZP therapy, mean ± SD, *p < 0.01 according to paired t test: DAS28(ESR) at each time points vs. baseline. **B** Classification of disease activity by the DAS28(ESR) and changes in disease activity at 12 weeks after CZP administration. Numbers represent percentages of all patients (%). **C** Left panel: changes in serum TNFα concentration up to 48 h after the CZP therapy, right panel: changes in serum IL-6 concentration up to 48 h after CZP initiation; data are shown as median (IQR). **D** Changes in serum CZP concentration up to 48 h after the CZP initiation; data are shown as mean ± SD

The continuation rate through 12 weeks of CZP treatment was 92% (Supplementary figure S1). The most common reason for discontinuation was poor response (*n* = 6, 6%). One patient discontinued CZP due to a serious infection (Supplementary Table S1). Significant improvement in the disease activity was observed at 24 h after initiation of CZP therapy, which was maintained until week 12 (Fig. 1A). At week 12, about 39% and 55% of patients treated with CZP achieved DAS28(ESR)<2.6 (remission) and <3.2 (low disease activity), respectively (Fig. 1B). Serum levels of both TNFα and IL-6 significantly decreased from baseline to 24 and 48 h after the first administration of CZP (Fig. 1C). The mean CZP concentration increased to 11.3 μg/mL and 24.2 μg/mL at 24 h and 48 h after the first administration of CZP (Fig. 1D).

### Baseline serum TNF-α levels and effectiveness of CZP at week 12 were not related

**Fig. 2:**
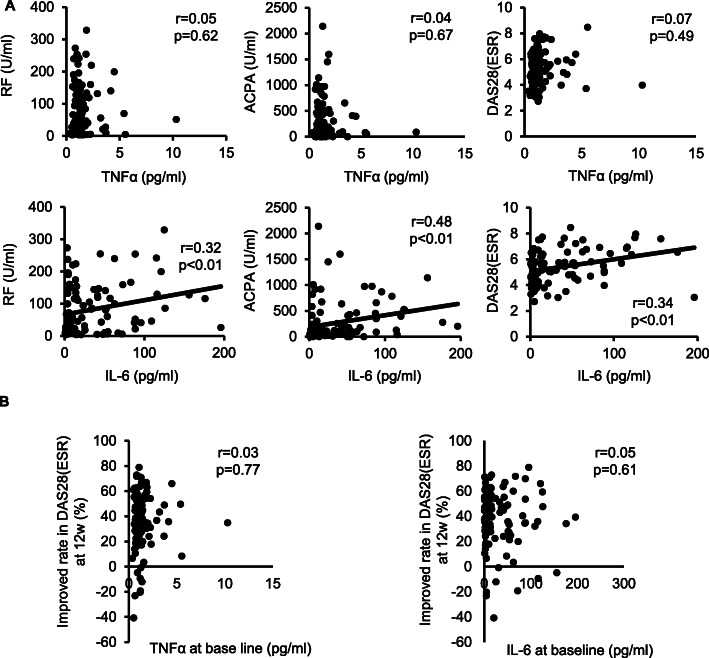
Relation between serum levels of TNF and IL-6 at baseline with RF, ACPA, DAS28(ESR), and changes in DAS28(ESR) from baseline to week 12. **A** Relation of serum levels of TNF and IL-6 at baseline to serum levels of RF and ACPA and DAS28(ESR) at baseline was assessed. **B** Relation of serum levels of TNF and IL-6 at baseline to %improvement of DAS28(ESR) from baseline to week 12 after CZP therapy was shown. DAS28(ESR) improvement rate (%) = [DAS28(ESR) at baseline - DAS28(ESR) at week 12]/DAS28(ESR) at baseline x100; All *p* values are according to Spearman’s rank correlation coefficient

Next, the statistical relationships of serum levels of TNFα and IL-6 at baseline to several clinical signs, laboratory test results, disease activity, and serum rheumatoid factor (RF) levels and anti-cyclic citrullinated peptide antibody (ACPA) were assessed. Serum IL-6 levels were correlated with more clinical parameters at baseline, including health assessment questionnaire-disability index (HAQ-DI), C-reactive protein (CRP), and ESR, than TNFα levels (Supplementary Table S2). Although serum IL-6 levels were significantly correlated with serum RF levels, ACPA and DAS28(ESR) at baseline, TNFα baseline levels was not correlated with them (Fig. 2A). Furthermore, serum levels of both TNFα and IL-6 were not correlated with an improvement in DAS28(ESR) from baseline to week 12 after the CZP therapy (Fig. 2B).

### Serum TNF-α levels at 24 h were negatively correlated with serum CZP levels at 24 h and effectiveness to CZP at week 12

**Fig. 3:**
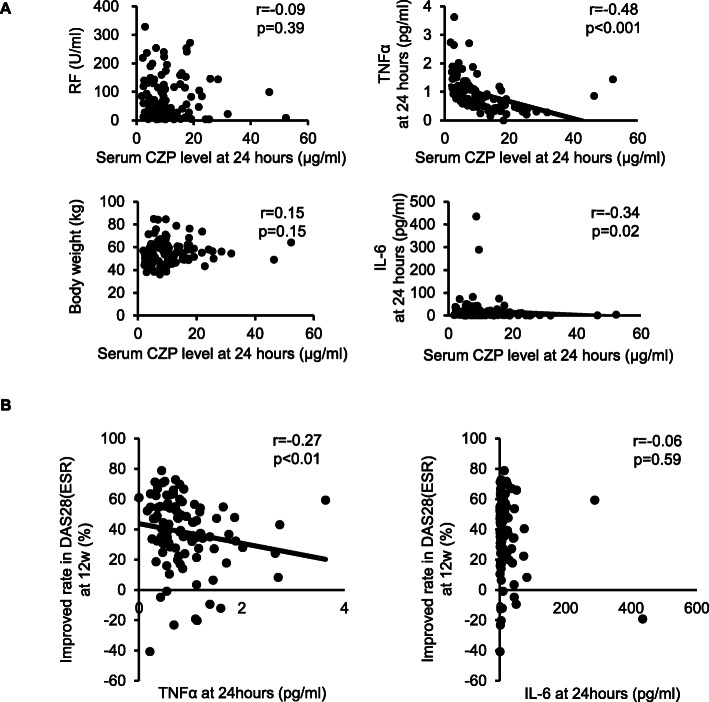
Correlation between serum levels of TNF and CZP at 24 h and negative correlation of TNF levels at 24 h and improvement in DAS28(ESR) from baseline to week 12. **A** Relation of serum levels of CZP at 24 h to RF, body weight at baseline, serum levels of TNF and IL-6 at 24 h after the CZP therapy was shown. **B** Relation of serum levels of TNF and IL-6 at 24 h to %improvement of DAS28(ESR) from baseline to week 12 after CZP therapy was shown. All *p* values are according to Spearman’s rank correlation coefficient

Serum levels of CZP at 24 or 48 h after administration were negatively correlated with age (CZP at 24 h, *r* = −0.38, *p* < 0.001) and ESR at baseline and positively correlated with Euroqol-5 dimension (EQ-5D), but they did not relate to the majority of clinical parameters at baseline (Supplementary Table S3). Serum CZP levels at 24 h did not relate to RF levels and body weight at baseline (Fig. 3A). In contrast, serum TNF-α levels at 24 h had a strong negative correlation with serum CZP levels at 24 h after administration (*r* = −0.48, *p* < 0.001), whereas those of IL-6 showed a weak negative correlation with CZP levels at 24 h (*r*= −0.34, *p* = 0.02) (Fig. 3A). Furthermore, serum TNF-α levels at 24 h was negatively correlated with improvement (%) in DAS28-ESR from baseline to week 12 after CZP therapy (*r* = −0.27, *p* < 0.01), whereas those of IL-6 were not related to improvement in DAS28-ESR (Fig. 3B).

To determine the correlation of DAS28-ESR < 2.6 at week 12 after CZP therapy with clinical parameters at study entry, univariate analysis of multiple variables was carried out. The univariate analysis showed that various factors were associated with the achievement of DAS28-ESR < 2.6, at week 12. Subsequently, multiple logistic regression analysis was performed using the factors with *p* < 0.05 in the univariate analysis after adjusting for confounding variables. Only serum TNF-α levels at 24 h had a negative correlation with the achievement of DAS28(ESR)<2.6 at week 12 after the CZP therapy in the multivariate analysis (odds ratio 0.01, 95% confidence interval 0.04e−2–0.22, *p* < 0.01) (Table 2). Patient characteristics at baseline that were correlated with TNFα levels at 24 h after the administration of CZP were identified by simple regression and multiple regression analyses (Supplementary Table S4). Simple regression analysis showed that baseline DAS28-ESR, HAQ-DI, EQ-5D, CRP, RF, and TNFα levels were associated with TNFα levels at 24 h after the administration of CZP. However, multiple regression analysis using these factors revealed that only baseline EQ-5D and TNFα levels were associated with TNFα levels at 24 h.

**Table 2:** Predictive factors for DAS28(ESR) <2.6 at week 12 identified by univariate and multivariate logistic regression analysis

	Univariate analysis		Multivariate analysis	
	Odds ratio (95% CI)	*P value*	Odds ratio (95% CI)	*P value*
Age	0.99 (0.95–1.02)	0.41		
RA duration	1.00 (0.99–1.00)	0.37		
MTX dose	1.07 (0.94–1.238)	0.30		
DAS28(ESR)	0.50 (0.31–0.75)	<0.001	0.67 (0.40–1.06)	0.09
HAQ-DI	0.69 (0.36–1.29)	0.25		
EQ-5D	41.89 (1.88–1496.64)	0.02	1.66 (0.01–287.19)	0.83
CRP	0.88 (0.73–1.03)	0.12		
RF	1.00 (0.99–1.00)	0.50		
ACPA	1.00 (0.99–1.00)	0.30		
MMP-3	1.00 (0.99–1.00)	0.69		
TNFα	1.18 (0.98–1.72)	0.09		
IL-6	1.00 (0.99–1.01)	0.46		
HAQ-DI at 24 h	0.53 (0.25–1.04)	0.07		
EQ-5D at 24 h	123.61 (4.82–5356.39)	<0.001	26.33 (0.514–7838.05)	0.09
CRP at 24 h	0.85 (0.66–1.02)	0.08		
CRP at 48 h	0.77 (0.53–1.02)	0.07		
TNFα at 24 h	0.20 (0.05–0.55)	<0.001	0.01 (0.04e−2–0.22)	<0.01
TNFα at 48 h	0.01 (2.636e-5–0.54)	<0.001	9.26 (0.30–342.47)	0.20
IL-6 at 24 h	0.98 (0.95–0.99)	0.03	1.00 (0.97–1.01)	0.92
IL-6 at 48 h	0.04 (0.01–0.60)	0.02	0.97 (0.92–1.03)	0.27
CZP at 24 h	15.56 (1.95–154.84)	<0.001	1.00 (0.99–1.01)	0.33
CZP at 48 h	16.76 (1.82–190.40)	0.01	1.00 (0.99–1.01)	0.24

*RA* rheumatoid arthritis, *MTX* methotrexate, *DAS* disease activity score, *HAQ-DI* health assessment questionnaire disability index, *EQ-5D* EuroQol 5 Dimension, *CRP* C-reactive protein, *RF* rheumatoid factor, *ACPA* anti-cyclic citrullinated peptide antibody, *MMP-3* matrix metalloproteinase 3, *TNFα* tumor necrosis factor, *IL-6* interleukin-6, *CZP* cerolizumab pegol

The rate of decrease in serum TNFα levels at 24 h after the administration of CZP is associated with DAS28-ESR remission at 12 weeks (odds ratio 1.02, 95% CI 1.01–1.03, *p* < 0.01). However, logistic regression analysis using DAS28-ESR remission at 12 weeks after the administration of CZP as an objective variable, and serum TNFα levels and the rate of decrease in serum TNFα levels at 24 h as explanatory variables showed that only low serum TNFα level at 24 h was associated with DAS28-ESR remission at 12 weeks (serum TNFα level at 24 h; odds ratio 0.22, 95% CI 0.06–0.82, *p* = 0.01; rate of decrease in serum TNFα level at 24 h; odds ratio 1.01, 95% CI 0.99–1.02, *p* = 0.25). In other words, DAS28-ESR remission at 12 weeks is more strongly associated with low serum TNFα levels at 24 h than with the rate of decrease in serum TNFα levels at 24 h.

### Serum TNF-α levels at 24 h after first administration of CZP predict CZP effectiveness

**Fig. 4:**
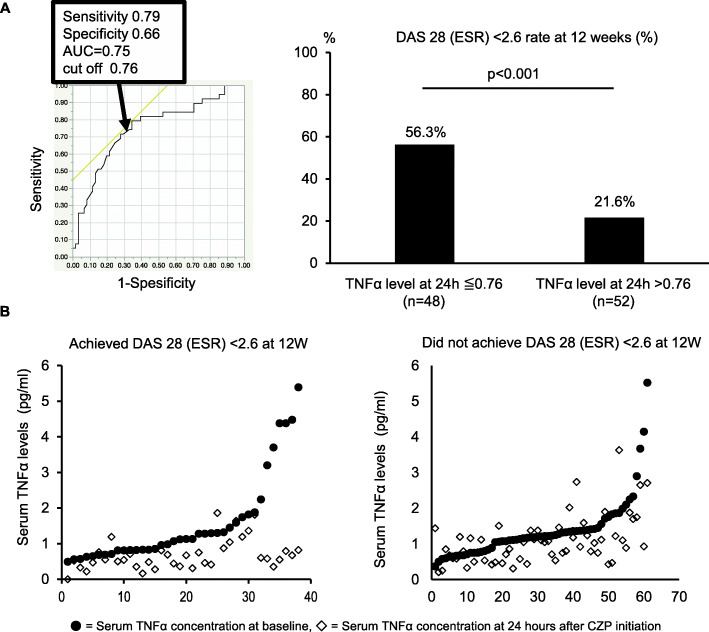
Disease activity at week 12 according to serum levels of TNF at 24 h after the CZP therapy: TNF-high vs. -low. **A** Left panel: ROC curve of serum TNFα concentration at 24 h after the first dose of CZP and the cut-off value to predict DAS28(ESR)<2.6 at week 12, right panel: comparison of DAS28(ESR) <2.6 rate at week 12 between the 2 groups based on whether the serum TNFα concentration at 24 h was above or below the cut-off value; *p* value according to Fisher’s exact test. **B** Left panel: cumulative probability plot of serum TNFα concentrations at baseline and 24 h in the group achieving DAS28(ESR)<2.6 at week 12; right panel: cumulative probability plot of serum TNFα concentrations at baseline and 24 h in the group not achieving DAS28(ESR)<2.6 at week 12, ● = serum TNFα concentration at baseline, ◇ = serum TNFα concentration at 24 h after CZP initiation

Based on these findings, a receiver operating characteristic (ROC) analysis was conducted to estimate the achievement of DAS28-ESR < 2.6 at week 12 after CZP therapy and a cut-off value of 0.76 pg/ml for serum TNF-α levels at 24 h was obtained (left panel of Fig. 4A). According to the cut-off value, DAS28-ESR < 2.6 was achieved at week 12 in 56.3% and 21.6% of patients with TNF-α levels ≤ 0.76 pg/mL and > 0.76 pg/mL at 24 h, respectively (*P* < 0.001, right panel of Fig. 4A).

Patient characteristics at baseline that were associated with TNFα levels of ≤ 0.76 at 24 h after the administration of CZP were identified by simple regression and multiple regression analyses (Supplementary Table S5). In the univariate analysis, younger patients and shorter disease durations at baseline showed a higher association with TNFα levels of ≤ 0.76 at 24 h after the administration of CZP. However, multivariate analysis did not identify any variable that was associated with TNFα levels of ≤ 0.76 at 24 h.

Figure 4B shows the cumulative probability plot of serum TNF-α levels at baseline and 24 h after the CZP therapy for the groups that did and did not achieve DAS28(ESR)<2.6 at week 12. Although the distribution of TNF-α levels at baseline was similar between the two groups, the distribution of TNF-α levels at 24 h was lower in the group that achieved DAS28-ESR < 2.6 than it was in the group that did not. These results indicate that CZP was highly effective in RA patients with low TNF-α concentrations 24 h after the initial administration.

## Discussion

In this study, which targeted biological DMARDs, we prospectively enrolled RA patients co-treated with CZP and MTX and analyzed the changes in effectiveness and biomarkers that occurred 24 h after CZP initiation. CZP was previously shown to be more effective than placebo at 1 week after the first dose was administered [11, 12], and in this study, we found that simple disease activity index decreased 24 h after administration of the first dose. Furthermore, CRP, ESR, and serum TNFα and IL-6 concentrations also decreased at 24 h after CZP initiation. CZP was effective at 24 h after the first dose and was extremely fast-acting.

There was no difference in the distribution of baseline TNF-α concentrations between the groups that achieved and did not achieve remission at week 12, and no correlation was found between baseline TNF-α concentration and improvement of disease activity. Numerous studies have investigated the use of baseline clinical signs in evaluating RA patients, such as serum response and serum cytokine concentrations, to predict the effectiveness of TNF inhibitor s[16–19]. Similar to our present findings, those studies found no relationship between baseline serum TNF-α concentration and the effectiveness of TNF inhibitors.

However, the RISING stud y[20] showed that a higher dose of infliximab was more effective in patients with high serum TNF-α concentration at baseline. In the C-OPERA stud y[21], which was also conducted in RA patients treated with CZP, higher baseline serum TNF-α concentrations were associated with higher rates of clinical remission with CZP at week 52. These studies highlighted the association between serum TNF-α concentration and the effectiveness of TNF-α inhibitors. The C-OPERA study was conducted in treatment-naïve patients with early-onset RA. The C-OPERA study, which was conducted in treatment-naïve patients with early onset RA reported a mean disease duration of 4 months, whereas in the present study it was 29 months.

MTX was not used at baseline in the C-OPERA study, but was administered at a median dose of 14 mg/w at baseline in this study. The relationship between serum TNF-α concentration and the effectiveness of TNF-α inhibitors showed differences between previous studies and the present study. These differences are likely due to factors influencing baseline serum TNF-α concentration such as disease duration, the DMARDs administered, and differences in disease activity. Patients have varying backgrounds in the real world, and therefore, baseline serum TNFα concentration may not be appropriate as a prognostic factor.

This study showed that lower TNF-α concentration at 24 h after CZP initiation was associated with higher chance of achieving remission at week 12. This strong relationship was presumably due to the absence of factors other than CZP that affected disease activity or serum TNF-α concentrations at 24 h after the first dose. To date, to the best of our knowledge, there have been no reports on the relationship between serum biomarkers measured the day after the first dose of a TNF-α inhibitor and its therapeutic effects, indicating the novelty of these findings. In RA patients affected by real-world factors, our findings suggest that the serum TNF-α concentration at 24 h after the first dose of CZP and not at baseline is useful in predicting the effectiveness of CZP treatment.

Factors that majorly contribute to serum TNFα levels at 24 h after CZP administration were identified from several clinical signs and serum biomarkers, namely age, sex, disease duration, rheumatoid factor, anti-CCP antibody, MMP-3, CRP, ESR, DAS28-ESR, HAQ, EQ-5D, BMI, serum TNFα level, serum IL-6 level, and serum CZP level, using the bootstrap forest method at baseline and 24 h. We found that CZP levels at 24 h showed the highest level of contribution and contribution rate (level of contribution 3.76, contribution rate 27.4%), and CZP levels at 24 h were significantly higher in patients whose serum TNFα levels at 24 h were lower than the cut-off value of 0.76 pg/mL than those whose serum TNFα levels were higher than the cut-off value (serum TNFα level ≤ 0.76 pg/mL, 14.3 ± 6.5; serum TNFα level > 0.76 pg/mL, 8.3 ± 9.0; *p* < 0.001). In Carron et al.’s pape r[22], it is reported that serum CZP levels after 24 h are inversely correlated with serum TNF and IL-6 levels. After 24 h, in Fig. 3A that have already accumulated in arthritic tissue at 5 h and may rapidly transition to the inflamed site, which leads to rapid onset of effect. This is probably because the stronger the level of arthritis, the higher the accumulation of CZP in tissues and lower its level in the serum.

Moreover, in the case that did not lead to remission (*n* = 21) despite the fact the serum TNFα concentration 24 h after the introduction of CZP was lower than the cut off value of 0.76 pg/ml 24 h after the introduction of CZP, serum IL-6 concentration 48 h after CZP introduction was significantly higher than those that resulted in a remission. This suggests that cases in which remission was not achieved despite lower TNFα concentrations after 24 h were more dependent on IL-6, and TNF inhibitors may have been ineffective. Prior to CZP administration, applying a sufficient amount of TNF inhibition to synovial membranes at higher levels in both TNF and IL-6 may narrow down cases with inflammatory synovial membranes dependent on IL-6.

The present study has some limitations that are worth mentioning. The response to CZP may be predicted with higher accuracy by factoring in cytokine levels and the genetic background of the patient, both of which were not analyzed in this study. Further studies are needed to analyze the addition of more cytokines and genetic background. In this study, we predicted the therapeutic response to CZP using factors that can be easily assessed clinically, and we conclude that TNFα levels at 24 h after CZP administration are clinically significant as an accurate predictor of the clinical response to CZP. No similar investigation has been conducted with other TNF-α inhibitors, and therefore, whether the onset of action at 24 h after the first dose and the association between the effectiveness and serum biomarkers at 24 h after the first dose are characteristics of CZP is still unknown. Previous studies have suggested that CZP may have higher potency for neutralizing TNF-α than that of adalimumab (ADA )[23, 24], and CZP tends to accumulate in inflammatory tissue s[22, 25]. Therefore, the effectiveness demonstrated at 24 h after the first dose may be unique to this drug. Because CZP is extremely fast-acting, it may be effective in patients whose serum TNF-α can be strongly neutralized at 24 h after CZP initiation, which may also be unique to CZP.

## Conclusions

In summary, the disease activity in RA patients started to improve 24 h after the first dose of CZP. The results of the present study also suggest that serum TNFα concentration at 24 h after CZP initiation may be used to predict the effectiveness at week 12. To increase the remission rate in RA, it is necessary to determine the effectiveness of the targeted synthetic DMARD used at an early point, in addition to how rapid the onset of action is. CZP is extremely fast-acting, and its effectiveness can be predicted as early as 24 h after the first dose, suggesting that it may be possible to determine the effectiveness early.

## Supplementary Information

**Additional files 1: Supplementary Figure S1.** Continuous rates for 12 weeks after CZP initiation as Kaplan-Meier curves. The continuation rate through 12 weeks of CZP treatment was shown.

**Additional files 2: Supplementary Table S1.** Reasons for discontinuing CZP. **Supplementary Table S2.** Relation of serum levels of TNF and IL-6 to clinical signs and laboratory data at baseline. **Supplementary Table S3.** Relation of serum CZP concentrations at 24 and 48 hours after the first administration to clinical signs and laboratory data at baseline. **Supplementary Table S4.** Predictive factors for serum TNFα levels at 24 hours identified by simple and multiple regression analysis. **Supplementary Table S5.** Predictive factors for serum TNFα levels <0.76 at 24 hours identified by identified by univariate and multivariate logistic regression analysis.

## Data Availability

The datasets used and/or analyzed during the current study are available from the corresponding author on reasonable request.

## Abbreviations

ACPA: Anti-cyclic citrullinated peptide antibody
CI: Confidence interval
CRP: C-reactive protein
CZP: Certolizumab pegol
DAS: Disease activity score
DMARD: Disease-modifying antirheumatic drug
EQ-5D: Euroqol-5 dimension
ESR: Erythrocyte sedimentation rate
GC: Glucocorticoids
HAQ-DI: Health Assessment Questionnaire-Disability Index
IL-6: Interleukin-6
MMP: Matrix metalloproteinase
MTX: Methotrexate
OR: Odds ratio
RA: Rheumatoid arthritis
RF: Rheumatoid factor
ROC: Receiver operating characteristic
TNFα: Tumor necrosis factor alpha
