# Supplementary material for: Serum TNFα levels at 24 h after certolizumab pegol predict effectiveness at week 12 in patients with rheumatoid arthritis from TSUBAME study
Source: Arthritis Res Ther. 2021 Jun 1;23:154. doi: 10.1186/s13075-021-02547-2 (PMC8167961; doi:10.1186/s13075-021-02547-2)
Supplement: Supplementary file 1 — Additional files 1: Supplementary Figure S1. Continuous rates for 12 weeks after CZP initiation as Kaplan-Meier curves. The continuation rate through 12 weeks of CZP treatment was shown. [file 13075_2021_2547_MOESM1_ESM.pptx]

## Slide 1
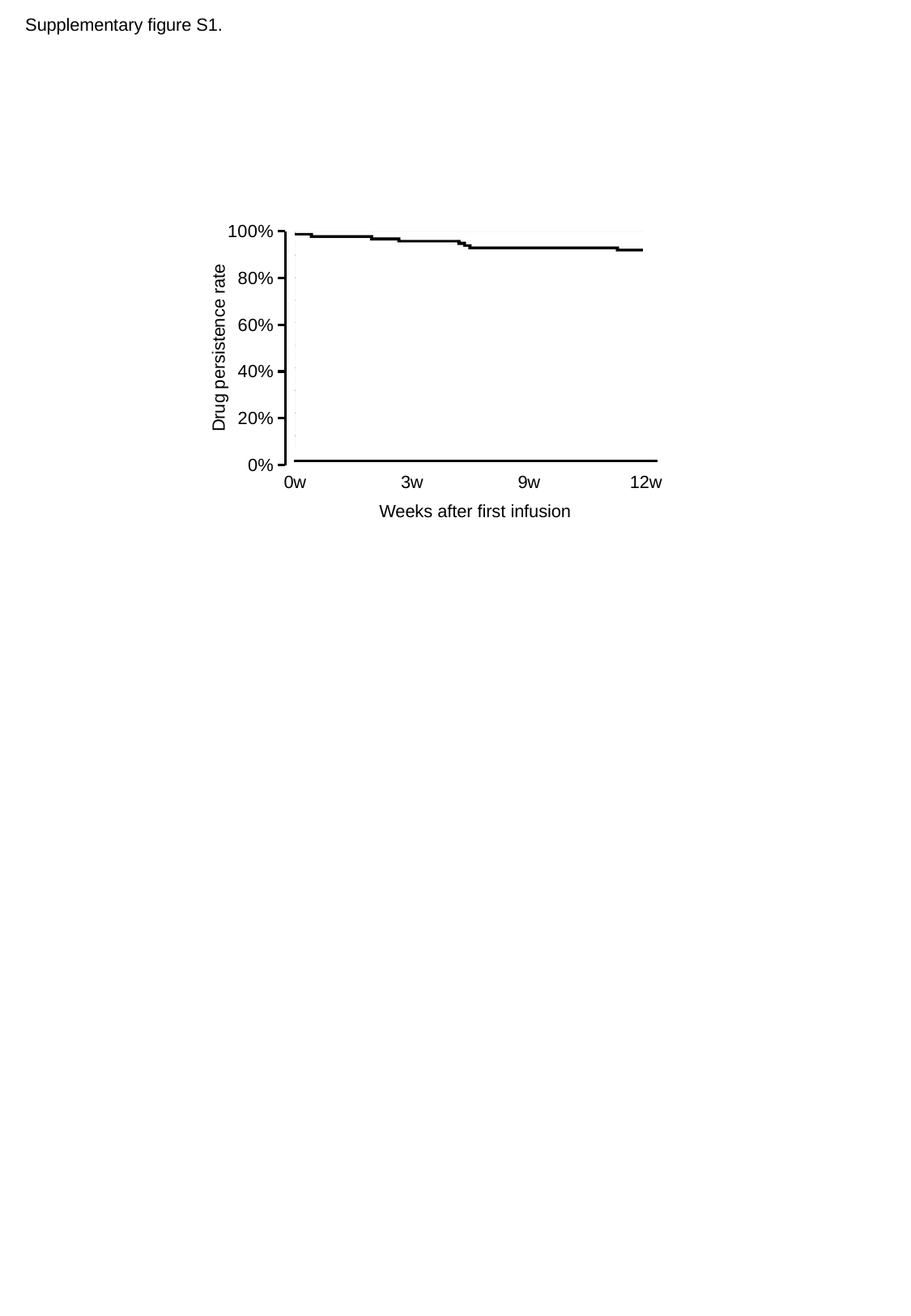

Supplementary figure S1.
### Chart
| Category | |
|---|---|
Drug persistence rate
0w
3w
9w
12w
Weeks after first infusion

## Slide 2
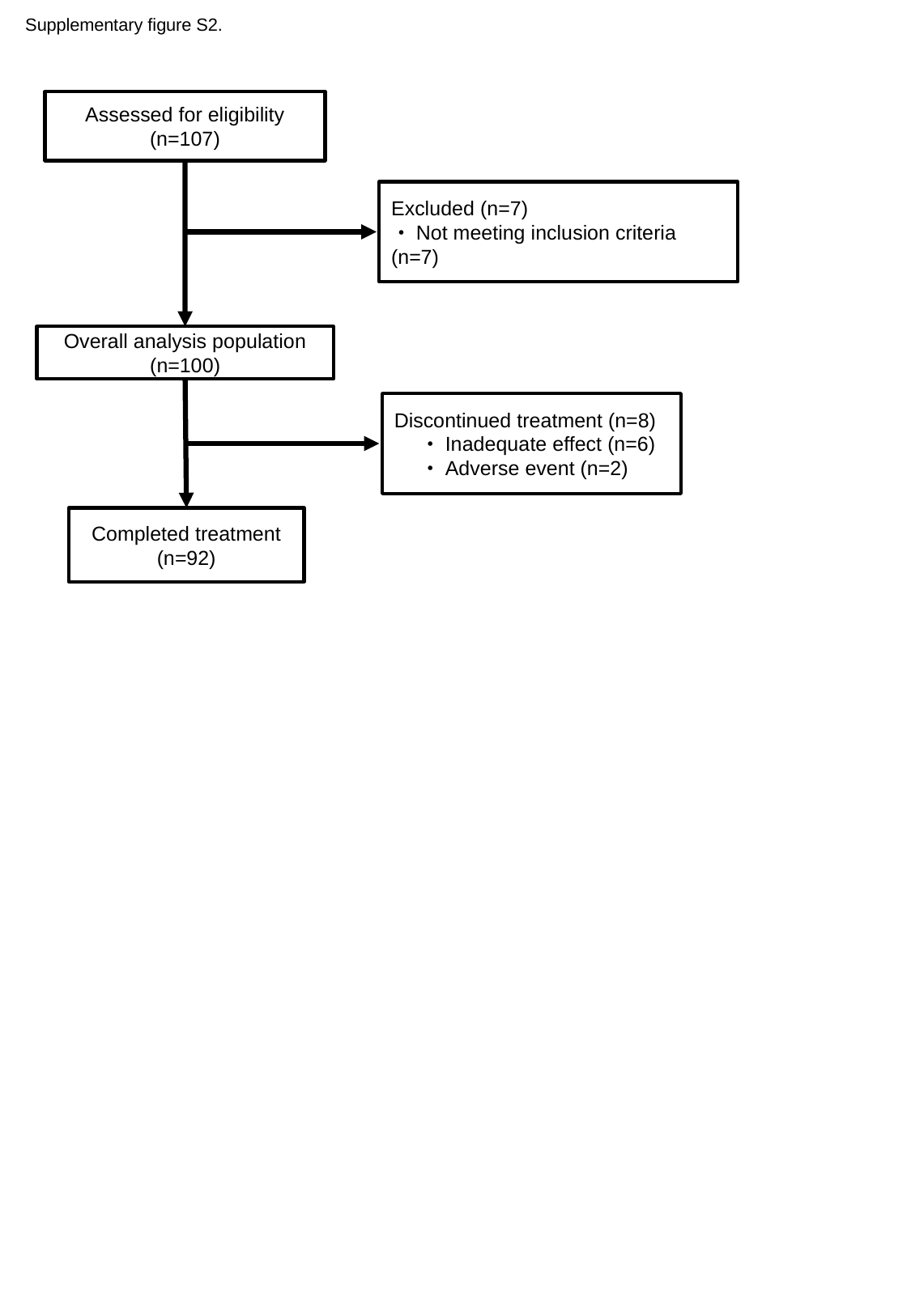

Supplementary figure S2.
Assessed for eligibility
(n=107)
Excluded (n=7)
・Not meeting inclusion criteria (n=7)
Overall analysis population (n=100)
Discontinued treatment (n=8)
 ・Inadequate effect (n=6)
 ・Adverse event (n=2)
Completed treatment (n=92)
