# Supplementary material for: Serum TNFα levels at 24 h after certolizumab pegol predict effectiveness at week 12 in patients with rheumatoid arthritis from TSUBAME study
Source: Arthritis Res Ther. 2021 Jun 1;23:154. doi: 10.1186/s13075-021-02547-2 (PMC8167961; doi:10.1186/s13075-021-02547-2)
Supplement: Supplementary file 2 — Additional files 2: Supplementary Table S1. Reasons for discontinuing CZP. Supplementary Table S2. Relation of serum levels of TNF and IL-6 to clinical signs and laboratory data at baseline. Supplementary Table S3. Relation of serum CZP concentrations at 24 and 48 hours after the first administration to clinical signs and laboratory data at baseline. Supplementary Table S4. Predictive factors for serum TNFα levels at 24 hours identified by simple and multiple regression analysis. Supplementary Table S5. Predictive factors for serum TNFα levels <0.76 at 24 hours identified by identified by univariate and multivariate logistic regression analysis. [file 13075_2021_2547_MOESM2_ESM.docx]

| Supplementary Table S1. Reasons for discontinuing CZP | |
| --- | --- |
| Events leading to discontinuation | n = 8 |
| Inadequate effect, n (%) | 6 (6%) |
| General malaise, n, (%) | 1 (1%) |
| Infection, n, (%) | 1 (1%) |

Supplementary Table S2. Relation of serum levels of TNF and IL-6 to clinical signs and laboratory data at baseline

|  |  | TNF-α  at base line | IL-6  at base line |
| --- | --- | --- | --- |
| Age (y) | r | 0.15 | 0.08 |
|  | p value | 0.13 | 0.45 |
| Disease duration (mo) | r | 0.08 | -0.28 |
|  | p value | 0.41 | 0.006 |
| MTX dose, mg/w | r | -0.01 | 0.05 |
|  | p value | 0.92 | 0.64 |
| 28-tender joint count | r | 0.07 | 0.02 |
|  | p value | 0.48 | 0.85 |
| 28-swollen joint count | r | 0.26 | 0.33 |
|  | p value | 0.006 | <0.001 |
| GH, VAS 0-100 mm | r | 0.06 | 0.18 |
|  | p value | 0.58 | 0.08 |
| EGA, VAS 0-100 mm | r | 0.26 | 0.33 |
|  | p value | 0.007 | <0.001 |
| DAS28-ESR | r | 0.19 | 0.34 |
|  | p value | 0.06 | <0.001 |
| HAQ-DI | r | 0.10 | 0.33 |
|  | p value | 0.31 | <0.001 |
| EQ-5D | r | -0.19 | -0.14 |
|  | p value | 0.06 | 0.16 |
| CRP (mg/dl) | r | 0.28 | 0.74 |
|  | p value | 0.007 | <0.001 |
| ESR (mm/h) | r | 0.18 | 0.61 |
|  | p value | 0.08 | <0.001 |
| Rheumatoid factor (U/ml) | r | 0.17 | 0.32 |
|  | p value | 0.10 | 0.001 |
| Anti-CCP antibody (U/ml) | r | 0.03 | 0.20 |
|  | p value | 0.75 | 0.04 |

MTX: methotrexate, bDMARDS: biological disease modifying anti-rheumatic drugs, GH VAS: patient's global assessment of disease activity visual analogue scale, EGA VAS: evaluator global assessment of disease activity visual analogue scale, DAS: disease activity score, HAQ-DI: health assessment questionnaire disability index, EQ-5D: EuroQol 5 Dimension, CRP: C-reactive protein, ESR: erythrocyte sedimentation rate

Supplementary Table S3. Relation of serum CZP concentrations at 24 and 48 hours after the first administration to clinical signs and laboratory data at baseline

|  |  | Serum CZP levels  at 24 h | Serum CZP levels  at 48 h |
| --- | --- | --- | --- |
| Age (y) | r | -0.38 | -0.21 |
|  | p value | <0.001 | 0.04 |
| Body weight (kg) | r | 0.15 | -0.01 |
|  | p value | 0.15 | 0.92 |
| Disease duration (mo) | r | -0.20 | -0.17 |
|  | p value | 0.05 | 0.09 |
| MTX dose, mg/w | r | 0.10 | 0.05 |
|  | p value | 0.32 | 0.60 |
| 28-tender joint count | r | -0.11 | -0.07 |
|  | p value | 0.27 | 0.47 |
| 28-swollen joint count | r | -0.19 | -0.15 |
|  | p value | 0.06 | 0.14 |
| GH, VAS 0-100 mm | r | 0.01 | 0.01 |
|  | p value | 0.98 | 0.91 |
| EGA, VAS 0-100 mm | r | -0.16 | -0.13 |
|  | p value | 0.12 | 0.21 |
| DAS28-ESR | r | -0.23 | -0.20 |
|  | p value | 0.03 | 0.05 |
| HAQ-DI | r | -0.08 | -0.03 |
|  | p value | 0.43 | 0.75 |
| EQ-5D | r | 0.02 | 0.01 |
|  | p value | 0.82 | 0.90 |
| CRP (mg/dl) | r | -0.13 | -0.13 |
|  | p value | 0.19 | 0.20 |
| ESR (mm/h) | r | -0.24 | -0.27 |
|  | p value | 0.02 | <0.01 |
| Rheumatoid factor (U/ml) | r | -0.09 | -0.05 |
|  | p value | 0.38 | 0.64 |
| Anti-CCP antibody (U/ml) | r | 0.01 | -0.01 |
|  | p value | 0.98 | 0.95 |

MTX: methotrexate, bDMARDS: biological disease modifying anti-rheumatic drugs, GH VAS: patient's global assessment of disease activity visual analogue scale, EGA VAS: evaluator global assessment of disease activity visual analogue scale, DAS: disease activity score, HAQ-DI: health assessment questionnaire disability index, EQ-5D: EuroQol 5 Dimension, CRP: C-reactive protein, ESR: erythrocyte sedimentation rate

Supplementary Table S4. Predictive factors for serum TNFα levels at 24 hours identified by simple and multiple regression analysis

|  | Simple regression analysis | | Multiple regression analysis | |
| --- | --- | --- | --- | --- |
|  | Regression coefficient  （95% CI） | P  value | Regression coefficient  （95% CI） | P  value |
| Age | 0.0064 (-0.0028 – 0.0156) | 0.17 |  |  |
| RA duration | 0.0006 (-0.0007 – 0.0019) | 0.42 |  |  |
| MTX dose | -0.0199 (–0.0536 - 0.0137) | 0.24 |  |  |
| DAS28 (ESR) | 0.1289 (0.0374 – 0.2204) | 0.006 | 0.0555 (-0.0654 – 0.1765) | 0.36 |
| HAQ-DI | 0.1787 (0.0194 – 0.3379) | 0.03 | -0.0960 (-0.3220 – 0.1299) | 0.40 |
| EQ-5D | -1.1771 (–1.8993- -0.4548) | 0.002 | -0.9222 (-1.7874 - -0.0569) | 0.04 |
| CRP | 0.0520 (0.0131 – 0.0909) | 0.009 | 0.0253 (-0.0218 – 0.0724) | 0.29 |
| RF | 0.0017 (0.0002 – 0.0032) | 0.03 | 0.0009 (-0.0006 – 0.0025) | 0.24 |
| ACPA | -0.0001 (-0.0003 – 0.0002) | 0.64 |  |  |
| MMP-3 | 0.0002 (-0.0002 – 0.0006) | 0.40 |  |  |
| TNFα | 0.1134 (0.0228 – 0.2040) | 0.01 | 0.0948 (0.0078 – 0.1818) | 0.03 |
| IL-6 | 0.0016 (-0.0013 – 0.0044) | 0.27 |  |  |

RA: rheumatoid arthritis, MTX: methotrexate, DAS: disease activity score, HAQ-DI: health assessment questionnaire disability index, EQ-5D: EuroQol 5 Dimension, CRP: C-reactive protein, RF: rheumatoid factor, ACPA: anti-cyclic citrullinated peptide antibody, MMP-3: matrix metalloproteinase 3, TNFα: tumor necrosis factor, IL-6: interleukin-6, CZP: cerolizumab pegol.

Supplementary Table S5. Predictive factors for serum TNFα levels <0.76 at 24 hours identified by identified by univariate and multivariate logistic regression analysis

|  | Univariate analysis | | Multivariate analysis | |
| --- | --- | --- | --- | --- |
|  | Regression coefficient  （95% CI） | P  value | Regression coefficient  （95% CI） | P  value |
| Age | 0.96 (0.93 – 0.99) | 0.03 | 1.00 (0.99 – 1.00) | 0.10 |
| RA duration | 0.99 (0.99 – 1.01) | 0.05 | 0.97 (0.94 – 1.00) | 0.07 |
| MTX dose | 1.08 (0.97 – 1.22) | 0.17 |  |  |
| DAS28 (ESR) | 0.84 (0.61 – 1.16) | 0.29 |  |  |
| HAQ-DI | 0.89 (0.52 – 1.52) | 0.66 |  |  |
| EQ-5D | 4.66 (0.35 – 62.64) | 0.25 |  |  |
| CRP | 0.90 (0.78 – 1.04) | 0.16 |  |  |
| RF | 1.00 (0.99 – 1.00) | 0.66 |  |  |
| ACPA | 1.00 (0.99 – 1.00) | 0.51 |  |  |
| MMP-3 | 1.00 (0.99 – 1.00) | 0.60 |  |  |
| TNFα | 0.81 (0.56 – 1.23) | 0.27 |  |  |
| IL-6 | 1.00 (0.99 – 1.01) | 0.39 |  |  |

RA: rheumatoid arthritis, MTX: methotrexate, DAS: disease activity score, HAQ-DI: health assessment questionnaire disability index, EQ-5D: EuroQol 5 Dimension, CRP: C-reactive protein, RF: rheumatoid factor, ACPA: anti-cyclic citrullinated peptide antibody, MMP-3: matrix metalloproteinase 3, TNFα: tumor necrosis factor, IL-6: interleukin-6, CZP: cerolizumab pegol.
